# Cytokine supplementation influences bovine embryo transcriptome during the preimplantation period

**DOI:** 10.1530/RAF-25-0011

**Published:** 2025-10-30

**Authors:** Katy S McDonald, Randall S Prather, M Sofia Ortega

**Affiliations:** ^1^ Division of Animal Sciences, University of Missouri, Columbia, Missouri, USA; ^2^ Department of Animal and Dairy Sciences, University of Wisconsin-Madison, Madison, Wisconsin, USA

**Keywords:** FGF2, LIF, IGF1, bovine embryo, embryo culture

## Abstract

**Abstract:**

The central goal of the following studies was to understand how FGF2, LIF, and IGF1, a cocktail called ‘FLI’, influence bovine embryo development by the degree of transcriptomic variation throughout preimplantation development. All embryos were produced *in vitro* with or without FLI supplementation at the beginning of culture. For each treatment, embryos were collected at the 4–6 cell, 9–16 cell, morula, or blastocyst stages, and RNA was isolated and sequenced at a depth of 50 million reads per sample. In the FLI group, at the 9–16 cell stage, there were seven upregulated and six downregulated differentially expressed genes (DEGs). At the morula stage, of the 1,856 DEGs, 580 were upregulated in FLI. Gene ontology analysis showed increased MAPK signaling, TGF-beta signaling, and Hippo signaling, which all help regulate cell adhesion, lineage commitment, and growth regulation in the developing embryo. In FLI blastocyst stage embryos, 199 upregulated and 545 downregulated DEGs revealed an increase in processes associated with interferon-gamma production and cell differentiation. Overall, FLI modulates many of the regulatory pathways in the developing embryo to drive increased cell survival, cell integrity, and overall embryo development.

**Lay summary:**

This study investigated whether adding three supportive proteins – FGF2, LIF, and IGF1 (together called FLI) – could improve the development of cow embryos grown *in vitro*. In cattle breeding, embryos are often produced outside the body to enhance fertility and support genetic selection. However, many embryos fail to develop properly under laboratory conditions. To address this, researchers tested whether FLI could create a more favorable environment for early embryo growth. Although embryos grown with and without FLI appeared similar under the microscope, gene expression analysis revealed important differences. Embryos exposed to FLI showed signs of improved cell survival, healthier growth, and reduced stress. These molecular changes suggest that FLI may help embryos become more resilient to key procedures such as freezing and transfer. The findings support the use of FLI as a culture supplement to improve the efficiency and success of *in vitro* embryo production systems used in livestock reproductive biotechnologies.

## Introduction

*In vitro* embryo production (IVP) is a useful tool in many species to improve fertility and enhance and preserve genetics (Hansen & Block 2004). It has a role in helping understand molecular and physiological events during preimplantation embryo development, as well as biomedical and agricultural applications. The use of advanced reproductive technologies such as IVP is growing each year but comes with limitations (Farin *et al.* 2001, Ealy *et al.* 2019, Viana 2021). Currently, less than 50% of IVP embryos will develop to the blastocyst stage (Luciano *et al.* 2018), and those blastocyst stage embryos have compromised viability following embryo transfer (Farin *et al.* 1999, Pontes *et al.* 2009, Bonilla *et al.* 2014, Ealy *et al.* 2019). An area of particular concern is the decreased survival observed with IVP embryos cryopreserved by slow-rate freezing (Farin *et al.* 1999, Pontes *et al.* 2009, Ferré *et al.* 2020).

One of the factors that influence embryo development and their ability to survive cryopreservation is the culture environment. There are differences between *in vivo* and *in vitro* environments, as well as between types of *in vitro* systems (Crosier *et al.* 2000, Lonergan *et al.* 2006). Culture conditions can induce alterations in embryo metabolism, development rate, mitochondria number, and epigenome. Reported divergence in gene message expression points to molecular differences in PI3K-Akt signaling, apoptosis, gap junction formation, and IFN-t production that lead to long-term effects such as impaired development, reduced pregnancy rate, and aberrant offspring (Neira *et al.* 2010, Noguchi *et al.* 2020). Given that the culture environment has such an effect on embryo development and survival, improving the medium to contain defined components without compromising viability will help improve consistency within the IVP system.

Supplementation of culture medium has been widely explored as a means of improving IVP and shifting toward a defined culture medium in multiple species (Sirisathien & Brackett 2003, Block *et al.* 2009, Neira *et al.* 2010, Fields *et al.* 2011, Mo *et al.* 2014, Yuan *et al.* 2017). Previous research described the positive effects of the addition of fibroblast growth factor 2 (FGF2), leukemia inhibitory factor (LIF), and insulin-like growth factor 1 (IGF1) (40 ng/mL, 20 ng/mL, 20 ng/mL, respectively) in a cocktail defined as ‘FLI’ into the porcine IVP system (Yuan *et al.* 2017, Yang *et al.* 2019, Procházka *et al.* 2021, Redel *et al.* 2021). The result was improved oocyte nuclear maturation, a two-fold increase in the efficiency of blastocyst production, and the first evidence that FLI influenced MAPK1/3 activation (Yuan *et al.* 2017).

In ruminant IVP, FLI supplementation improved development to the blastocyst stage, as well as other quality parameters in the oocyte, such as ATP content, glutathione (GSH) levels, mitochondrial membrane potential, and reactive oxygen species (ROS) levels (Tian *et al.* 2021, Zhang *et al.* 2023). In a previous study from our group, FLI supplementation at the beginning of embryo culture had marked effects throughout the preimplantation period, improving development to the blastocyst stage from 31 to 42%, as well as *in vitro* re-expansion following slow-rate cryopreservation from 39 to 82%, with improved cytoskeleton integrity and reduced post-thaw apoptosis (Stoecklein *et al.* 2021).

In a follow-up study, slow-rate frozen embryos that were treated or not with FLI at the beginning of culture were transferred to synchronized females to evaluate post-transfer survival (Stoecklein *et al.* 2022). The supplementation of FLI during embryo culture did not increase conceptus recovery; nevertheless, day 15 conceptus transcriptome was affected by FLI supplementation during the preimplantation period. Biological and molecular functions related to conceptus and placental development, as well as maternal–embryo crosstalk, were increased in FLI-supplemented embryos. Among the increased genes in FLI-supplemented conceptuses were pregnancy-associated glycoproteins (PAGs) 2 and 12 (Stoecklein *et al.* 2022), which are distinct from the mononucleated trophectoderm cells (Davenport *et al.* 2024).

In addition, our group tested this FLI supplementation strategy on pregnancy success (McDonald *et al.* 2025). Pregnancy rates by day 32 were similar between control and FLI-supplemented embryos; however, pregnancies from FLI-supplemented embryos had increased circulating concentrations of pregnancy-associated glycoproteins by day 24 of gestation, which is in agreement with the day 15 study from Stoecklein *et al.* (2022). Thus, while embryos supplemented with FLI may appear phenotypically similar to their control counterparts, differences at the molecular level exist, resulting in more embryos available for transfer.

The addition of FLI to the culture medium increases the number of embryos that reach the blastocyst stage and are eligible for cryopreservation (Stoecklein *et al.* 2021), but the mechanisms that regulate these changes represent a gap in our knowledge. The objective of this study was to investigate FLI modulation of bovine embryo development throughout the first 7 days of *in vitro* culture. This study aimed to gain knowledge of *in vitro* embryo development and mechanisms that regulate early embryo development, as this is crucial for full adoption of the technology and optimization of protocols for embryo culture and cryopreservation.

## Materials and methods

**Figure 1:**
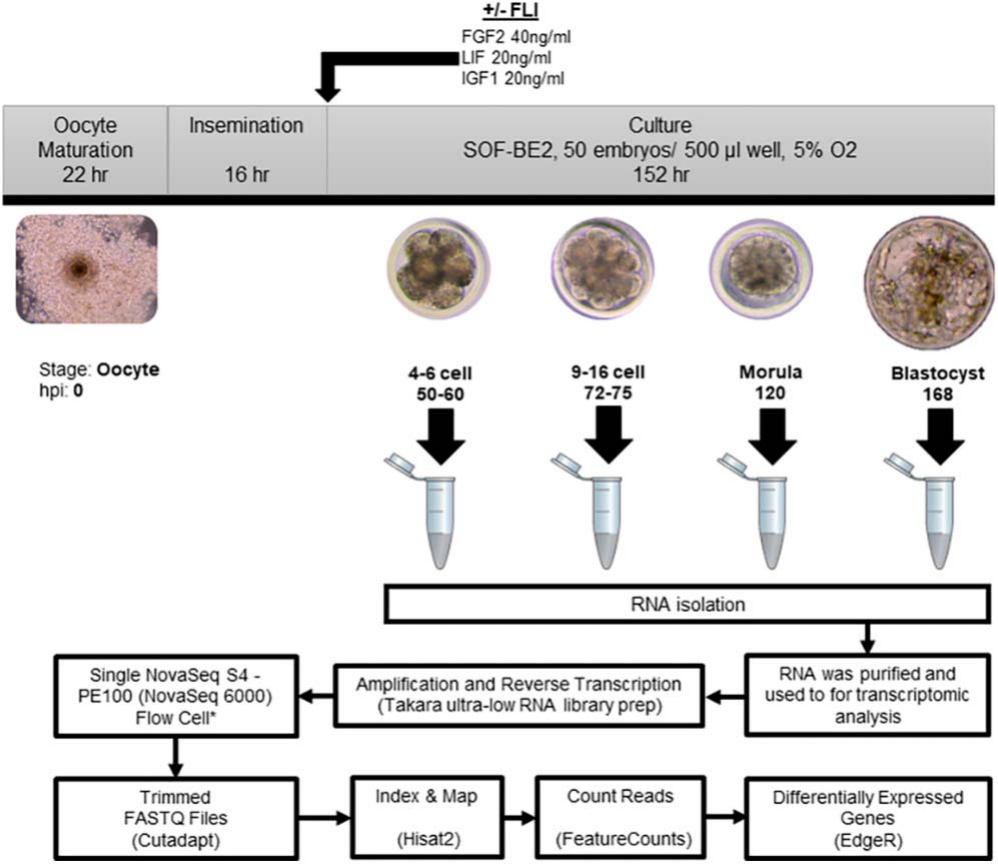
Experimental design. Abattoir-derived oocytes were matured for 22 h before fertilization for 16–18 h, and then placed in culture with or without (control) FLI. For each treatment, groups of fifteen embryos were collected at the 4–6 cell, 9–16 cell, morula, and blastocyst stages. RNA was isolated, and amplification and reverse transcription were performed. Libraries of cDNA were sequenced at a depth of 50 million reads per sample. After quality control, the transcriptome of samples was aligned to the cow genome by using Hisat2, and FeatureCounts was used to determine the read counts per gene. EdgeR was used to identify DEGs (FDR < 0.05).

The experimental design is presented in Fig. 1.

### Embryo production media

All embryo production media – oocyte maturation (OMM), fertilization (IVF-TALP), wash (HEPES-TALP), culture (SOF-BE2), and the supplements penicillamine, hypotaurine, and epinephrine (PHE) – were prepared in-house following recipes previously published (Tríbulo *et al.* 2019, Stoecklein *et al.* 2021). Sperm purification gradient Isolate® (Irvine Scientific, USA) was acquired ready to use. The cytokine cocktail, FLI, was supplemented as FGF2 (40 ng/mL, source: human FGF2 (made in-house)), LIF (20 ng/mL, source: human LIF (Millipore, USA; Catalog no. LIF1050)), and IGF1 (20 ng/mL, source: human IGF1 (Peprotech, USA; Catalog no. CYT-022)) to the culture medium (Yuan *et al.* 2017, Stoecklein *et al.* 2021). To achieve the desired concentration, 1.2 μL of the FLI cocktail was added to each treatment well of 500 μL SOF-BE2.

### Embryo production

Embryos were produced *in vitro* using abattoir-derived ovaries from Bos taurus cattle. Cumulus-oocyte complexes (COCs) were fertilized and cultured using standard procedures (Ortega *et al.* 2018, Tríbulo *et al.* 2019, Stoecklein *et al.* 2021). Briefly, COCs were collected from ovaries and placed in 500 μL of OMM (50 COCs/well) overlaid with 300 μL mineral oil and matured for 22–24 h in a humidified atmosphere containing 5% (v/v) CO_2_. At the end of maturation, the COCs were inseminated with sperm from a single Holstein bull known to have high fertility *in vitro* (Stoecklein *et al.* 2021). Fertilization proceeded for 18–20 h in a humidified atmosphere containing 5% (v/v) CO_2_. At the end of fertilization, putative zygotes (oocytes exposed to sperm) were denuded of their cumulus cells by vortexing for 5 min in 200 μL of hyaluronidase (10,000 units/mL) (Tríbulo *et al.* 2019). A minimum of 25 and up to 50 putative zygotes were placed in a 5-well dish with each well containing 500 μL of SOF-BE2 overlaid with 300 μL mineral oil. Half of the putative zygotes were supplemented with FLI at the moment they were placed in the culture plate. Embryos were cultured at 38.5°C in a humidified atmosphere containing 5% (v/v) O_2_ and 5% (v/v) CO_2_ with the balance N_2_.

### Embryo collection and RNA isolation

For each treatment, groups of fifteen embryos were collected at the 4–6 cell, 9–16 cell, morula, or blastocyst stages. This was repeated for four replicates. At the time of collection, embryos were placed in 0.1% (w/v) pronase for 60 s to degrade any cells outside of the zona pellucida. Embryos were then washed in nuclease-free PBS-PVP and snap-frozen in liquid nitrogen. Samples were stored at −80°C until RNA isolation using the PicoPure™ RNA Isolation Kit (ThermoFisher, USA). RNA isolation followed the manufacturer’s instructions, apart from using Nano spin columns (Luna Nanotech, Toronto, Ontario, CA, USA) instead of the mini columns provided. RNA was stored at −80°C until transcriptomic analysis was performed at the University of Missouri Genomics Technology Core. Total RNA concentration was determined using a Qubit HS RNA assay by Qubit fluorometer (Invitrogen, USA). The RNA integrity was confirmed with an Agilent fragment analyzer using the HS RNA kit. The average RNA concentration and integrity for each group are presented in Table 1.

**Table 1:** Average RNA concentration, 28s/18s, and RQN for samples submitted for RNA sequencing.

Stage/treatment	Concentration (ng/µL)	28s/18s	RQN
4–6 Cell			
Control	0.7	0.8	6.6
FLI	2.6	0.8	6.3
9–16 Cell			
Control	0.8	0.6	6.5
FLI	0.6	0.6	6.1
Morula			
Control	2.1	1.8	9.0
FLI	1.0	1.9	9.9
Blastocyst			
Control	1.6	2.0	9.8
FLI	1.2	2.2	9.2

### Transcriptome analysis

Amplification and reverse transcription were performed using the Takara ultra-low RNA library preparation method with reagents supplied in the Takara SMART-seq v4 Ultra Low-input RNA Kit (Takara Bio, USA). Libraries of cDNA were sequenced on a single NovaSeq S4 – PE100 (NovaSeq 6000) Flow Cell (NGS platform of Illumina). Sequencing was done at a depth of 50 million reads per sample. Following sequencing, the FASTQC tool (https://www.bioinformatics.babraham.ac.uk/ projects/fastqc/) was used to assess the quality of the fastq files. Using cutadapt version 1.18, FASTQ files were 3′ trimmed for Illumina adapters, for ambiguous nucleotides (N’s), and for poly-G artifacts resulting from Illumina two-color chemistry for reads whose 3′ ends overlapped with the adapter for a minimum of three bases with 90% identity (Martin 2011). After trimming, reads containing fewer than ten bases were discarded. The sample transcriptomes were aligned to the cow genome (Bos_taurus.ARS-UCD1.2.104) using Hisat2, and FeatureCounts was used to determine the read counts per gene (Liao *et al.* 2014, Kim *et al.* 2015, Rosen *et al.* 2020). Message expression analysis was performed using the Bioconductor package EdgeR, and a false discovery rate (FDR) of less than 0.05 was used to determine differentially expressed genes (DEGs) (Robinson *et al.* 2010, McCarthy *et al.* 2012, Chen *et al.* 2016).

Functional annotation of DEGs was performed using the DAVID (database for annotation, visualization, and integrated discovery) bioinformatics database (Huang *et al.* 2009*a*,*b*). This software provides an integrated biological knowledge base for efficiently identifying biological processes, patterns, and themes in large gene lists (Huang *et al.* 2009*a*). Ensembl gene IDs associated with DEGs were submitted to the DAVID analysis tools. The *Bos taurus* genome was used as the background gene list to provide identification for gene families that were identified in the over- and under-represented terms. In addition, gene ontology (GO) enrichment analysis was performed using ShinyGO 0.76 (Ge *et al.* 2020). This software is used to complement existing tools by producing KEGG pathway diagrams, hierarchical clustering trees, networks summarizing overlapping terms/pathways, protein–protein interaction schemes, gene characteristic plots, and enriched promoter motifs (Ge *et al.* 2020). Gene IDs were submitted and analyzed against the cow (*Bos taurus*) genome to generate enriched GO terms and represented pathways.

## Results

### Effect of FLI supplementation at 4–6 and 9–16 cell stages

**Figure 2:**
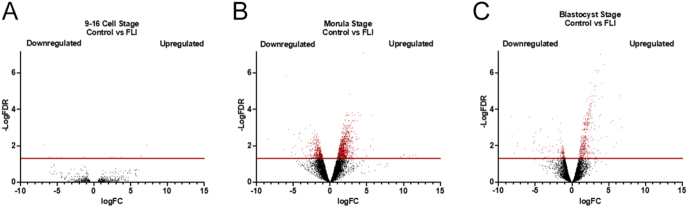
Volcano plots representing the number of DEGs when comparing embryos from FLI or control embryos. At a FDR < 0.05, there were (A) 13 DEGs identified at the 9–16 cell stage, (B) 1,856 DEGs detected at the morula stage, and (C) 744 DEGs at the blastocyst stage.

At the earliest (4–6 cell) stage, the average total transcript reads for the control treatment was 32,188,953, with an alignment to the bovine genome of 81.5%. For the embryos supplemented with FLI, the average total transcript reads was 32,170,548, with an alignment to the bovine genome of 82.6% (Table 2). At FDR ≤ 0.05, no DEGs were detected. At the 9–16 cell stage, the average total transcript reads for the control treatment was 17,982,989, with an alignment to the bovine genome of 79.9%. For the FLI treatment, the average total transcript reads was 41,001,645, with an alignment to the bovine genome of 79.7% (Table 2). At the 9–16 cell stage, 14 DEGs were detected at FDR ≤ 0.05 (Fig. 2A). Of these, eight were up-regulated in the FLI treatment: *SOX3, CAPN6, TENT5A, KCNB2, LRCH2, MGLL, GNG14,* and *E1BM77_BOVIN*. Moreover, downregulated genes in the FLI treatment were *CYP11A1, ISG15, USP18, KEL, G3MZI4_BOVIN,* and *BEX2*.

**Table 2:** Summary of the total transcript reads and aligned proportions to the bovine genome for embryos treated with or without FLI.

Stage/treatment	Transcript reads	Alignment	Differentially expressed	Comparison of FLI to control	
				Upregulated	Down-regulated
4–6 Cell			0		
Control	32,188,953	81.5%			
FLI	32,170,548	82.6%			
9–16 Cell			14	8	6
Control	17,982,989	79.9%			
FLI	41,001,645	79.7%			
Morula			1,856	580	1,276
Control	51,649,755	68.4%			
FLI	56,725,819	83.5%			
Blastocyst			744	199	545
Control	75,441,542	91.0%			
FLI	95,244,668	94.2%			

### Effect of FLI supplementation at morula and blastocyst stages

At the morula stage, the average total transcript reads for the control treatment was 51,649,755, with an alignment to the bovine genome of 68.4%. For the FLI treatment, the average total transcript reads was 56,725,819, with an alignment to the bovine genome of 83.5% (Table 2). At the morula stage, 1,856 DEGs were detected at FDR ≤ 0.05. Of these, 580 were up-regulated in the FLI treatment, while 1,276 were down-regulated (Fig. 2B). At the blastocyst stage, the average total transcript reads for the control treatment was 75,441,542, with an alignment to the bovine genome of 91.0%. For the FLI treatment, the average total transcript reads were 95,244,668, with an alignment to the bovine genome of 94.2% (Table 2). At the blastocyst stage, 744 DEGs were detected at FDR ≤ 0.05. Of these, 199 were increased in the FLI treatment, while 545 were decreased (Fig. 2C).

### GO ontology and KEGG pathway analysis

DAVID bioinformatics database and ShinyGO were used to identify biological processes associated with message families that were different between the two treatments. The top biological processes for morula and blastocyst stage embryos are summarized in Supplemental data sheets S1 and S2 (see section on Supplementary materials given at the end of the article).

At the 9–16 cell stage, embryos cultured in the presence of FLI show an over-representation of biological terms associated with transmembrane and membrane components. Terms that were under-represented in the FLI treatment are messages associated with IFN-stimulated genes and interferon-mediated signaling pathways.

Once the embryos reached the morula stage, an increase in DEGs was observed. These messages were enriched in functions including protein-containing complex assembly, autophagy, cell adhesion, and growth regulation. Biological terms such as proteoglycan biosynthesis, angiogenesis, TGF-beta signaling, integrin signaling, proliferation, and MAPK signaling were over-represented when FLI was supplemented into the culture medium. Conversely, in the control group, processes such as oxidative phosphorylation, metabolic pathways, mitochondrial function, and electron transport were increased.

**Figure 3:**
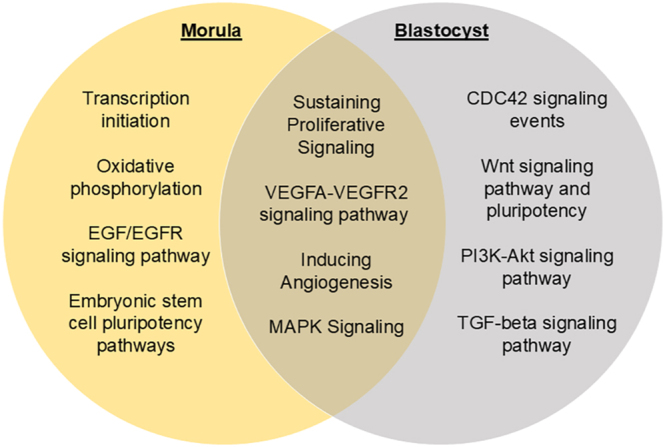
Venn diagram depicting the top pathways influenced by FLI supplementation at the morula and/or blastocyst stage.

At the blastocyst stage, there was an increase in messages associated with broad biological processes such as mitochondrial function and matrix, mitotic cell cycle, DNA-directed RNA polymerase, and DNA repair. WNT signaling and closely related cell migration were under-expressed in the FLI group compared to the control. In addition, zinc finger, protein binding, and regulation of transcription by RNA polymerase were also under-expressed. Overlapping biological patterns or mechanisms in the morula and blastocyst stage embryos include angiogenesis, vascular endothelial growth factor (VEGF) signaling, sustaining proliferative signaling, and MAPK signaling (Fig. 3).

## Discussion

Quality parameters that define the competency of an IVP embryo to survive cryopreservation and establish a pregnancy are not well defined and are frequently based on visual assessment (Abe *et al.* 1999, Crosier *et al.* 2000, Fair *et al.* 2001). As modifications are made to the IVP culture medium, it is important to understand how the embryo is affected beyond the phenotype (Melka *et al.* 2010, Driver *et al.* 2012, de Lima *et al.* 2020, Santos *et al.* 2021). The present study characterized how supplementing culture medium with FLI changed the transcriptome and the activation of several signaling pathways during preimplantation development in bovine embryos.

The first stage evaluated was 4–6 cell stage embryos at 50–60 h post-insemination. At this early time point, the bovine embryo genome is generally quiescent as it is still under the control of maternal factors (Frei *et al.* 1989). While minor genome activation may occur as early as the 4-cell stage, it is not until the 8-cell stage that major genome activation occurs (Graf *et al.* 2014). In this study, no DEGs were detected at the 4-cell stage. Perhaps, since the embryos have yet to degrade maternal RNAs, the differences, if any, caused by FLI at this stage do not surpass the transcript abundance already present in the oocyte (Bogliotti *et al.* 2020). Previous work from our group has already shown differences in gene expression at the 4-cell stage under other treatments (Lockhart *et al.* 2023), providing evidence that large treatment effects can be detected early in development.

Differences at the level of the transcriptome due to FLI were observed at later stages in preimplantation embryo development (9–16 cell, morula, and blastocyst stages). At the 9–16 cell stage, there were just 13 DEGs. These messages were involved in processes such as membrane integrity, peptidase activity, interferon signaling, and transmembrane components. Message expression for two genes, ISG15 ubiquitin-like modifier (*ISG15*) and ubiquitin-specific peptidase 18 (*USP18*), was lower in the FLI group compared to the controls. Both messages are involved in the interferon signaling cascade and embryo-induced changes in the uterus during early pregnancy in cattle; however, before the embryo entering the uterus, little is known about the roles of these gene messages (Bauersachs *et al.* 2006, Maillo *et al.* 2015). It is known that *ISG15* mRNA is present in the oocyte and early stages of embryo development, disappears by day 4 of development, and expression increases at the blastocyst stage to regulate interferon tau (IFNT) (Zhao *et al.* 2017). The variation in expression between the two groups could be explained by a deviation in development in the FLI treatment compared to controls. Embryos treated with FLI may have already entered the period of *ISG15* quiescence at this time in development.

Furthermore, expression for monoglyceride lipase (*MGLL*) was increased in the FLI group compared to the controls. This message encodes the enzyme that catalyzes the conversion of triglycerides to free fatty acids and glycerol. It has an important role in lipid metabolism and availability, and is associated with PI3K-AKT, an important pathway in early embryo development (Zhang *et al.* 2021). In the bovine embryo, activation of PI3K-AKT by IGF-1 has been reported to block heat shock-induced cellular apoptosis (Jousan *et al.* 2008). Another study explored message expression in embryos exposed to dietary long-chain fatty acids and found α-linolenic acid-exposed embryos had elevated *MGLL* and were less likely to degenerate (Salehi *et al.* 2016). Dysfunction in lipid metabolism and uptake in the early embryo could alter its viability, which has been widely studied in bovine embryos produced *in vitro* and *in vivo* (Mucci *et al.* 2006). It is possible, then, that the differences in expression between the two groups, associated with interferon signaling, PI3K signaling, and lipid regulation, lead to differences in viability and transcriptome observed later in development (Stoecklein *et al.* 2021, 2022).

As the embryo continues to develop, cells continually divide, undergo compaction, and a morula is formed (Soom *et al.* 1997). At the morula stage, the FLI group had increased message expression for genes associated with proteoglycan biosynthesis, angiogenesis, and signaling pathways such as mitogen-activated protein kinase (MAPK), transforming growth factor-β (TGF-β), and VEGF. These active signaling cascades in the embryo are associated with cell adhesion, lineage commitment, and growth regulation (Luo *et al.* 2002, Kuijk *et al.* 2012, Li *et al.* 2012, Anchordoquy *et al.* 2015). It is logical that MAPK would be increased in the FLI group, as the cytokines that compose the FLI cocktail, FGF2, LIF, and IGF1, activate that cascade after binding to their respective receptors. Fibroblast growth factors drive the expression of GATA6 through MAPK signaling, while LIF binding to glycoprotein 130 (gp130) activates MAPK and mediates cell differentiation (Neel *et al.* 2003, Graf *et al.* 2011, Yang *et al.* 2011). Binding of IGF1 to the IGF1 receptor stimulates MAPK and PI3K to support embryo development and survival (Matsui *et al.* 1997, Byrne *et al.* 2002). The Hippo signaling cascade was also increased in the FLI group compared to controls. During embryogenesis, Hippo signaling is activated by increased cell contact and cell polarity. This activation begins a cascade of phosphorylation events that eventually leads to YAP localization to the nucleus in differentiated trophectoderm cells (Negrón-Pérez & Hansen 2018, Sharma & Madan 2020). Differences at the level of mRNA processing were also observed. An increase in mRNA transport is especially important, as the localization of mRNA is key when cells divide and commit to their cell lineages (Pfeffer 2019). Taken together, FLI supplementation may increase or accelerate the activation of regulatory pathways such as MAPK, TGF-β, and PI3K-AKT in the bovine embryo.

**Figure 4:**
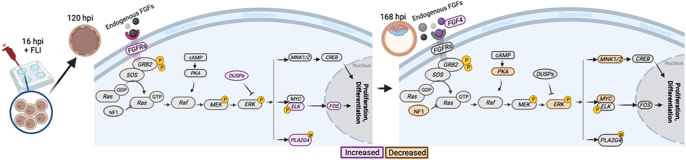
Graphical depiction of the classical map kinase (MAPK) pathway and its proposed signaling cascade in the morula and blastocyst stage embryos. At each stage, the genes representing messages that were increased or decreased (FDR < 0.05) in the FLI-supplemented group are indicated. At the morula stage, an increase in FGFR expression leads to an increase in DUSPs, negative regulators of the ERK pathway. The downstream increase in ELK and subsequently FOS drives proliferation and differentiation at this stage. At the blastocyst stage, an increase in endogenous FGF4 drives a signaling cascade characterized by a decrease in both PKA and ERK, possibly driven by the increase in DUSPs at the earlier stage. The decrease in ERK leads to decreased expression of both MNK and MYC, but no difference in CREB or FOS, both of which drive proliferation and differentiation of the cells.

Once the embryo reaches the blastocyst stage, it has a highly active metabolism, cell proliferation and differentiation are constant, and the embryo is preparing to hatch from the zona pellucida (Akizawa *et al.* 2016, Negrón-Pérez & Hansen 2017, de Lima *et al.* 2020). At this stage, 744 DEGs were detected between the control and FLI group, and they were associated with broad biological processes such as regulation of cell differentiation, enzyme binding, regulation of development, and metabolic processes. There was an increase in the message for interferon gamma production in the FLI-treated embryos. Interferon gamma is a type two proinflammatory interferon with a role in early pregnancy in many species; however, its effects in ruminant early pregnancy are unclear (Lefèvre *et al.* 1990, Ozornek *et al.* 1997, Cencic & La Bonnardière 2002). Like at the morula stage, MAPK signaling at the blastocyst stage was affected by FLI supplementation. Messages for four DEGs involved in the MAPK cascade were upregulated, while 20 were downregulated in the FLI group. Figure 4 summarizes the MAPK cascade and transcripts that differed between the two groups. In the FLI group, an increase in MAPK components *FGFR*, *ELK*, and *FOS* drives increased proliferation and differentiation at the morula stage. However, at the blastocyst stage, decreased *PKA* and *ERK* are observed, a possible effect of increased *DUSP*s observed at the earlier stage. Decreased *ERK* leads to reduced message expression of *MYC* and *MNK*1/2 in blastocyst-stage FLI embryos. The MAPK pathway is a highly conserved signaling mechanism in the early embryo; however, variance from species to species exists (McLean *et al.* 2014). In the bovine, MAPK inhibition does not inhibit development to the blastocyst stage but rather alters the ratio within the cell lineages (hypoblast, epiblast, and trophoblast) (Madan *et al.* 2005, McLean *et al.* 2014). Supplementation of FLI does not alter the ratio of inner cell mass to trophectoderm; however, further research is needed to understand if differences between hypoblast, epiblast, and trophoblast ratios exist (Stoecklein *et al.* 2021). The WNT signaling family of growth factor–receptor interactions, involved in numerous developmental processes, was decreased in the FLI group. These cascades require a balance of canonical and non-canonical signaling to promote cell proliferation, pluripotency maintenance, and cell migration (Denicol *et al.* 2013, Sidrat *et al.* 2020, Warzych *et al.* 2020). It has been reported that activation of canonical WNT signaling by day 5 in the preimplantation bovine embryo inhibits embryonic development at later stages (Denicol *et al.* 2013). More recently, it has been shown that the canonical Wnt/β-catenin pathway in coordination with PPARδ signaling is important for the regulation of bovine embryonic development (Sidrat *et al.* 2020). Others have reported that embryo-derived WNTs participate in the formation of the inner cell mass but are dispensable for blastocyst formation; however, maternally derived WNTs have both negative and positive effects depending on the WNT ligand (Tribulo *et al.* 2017). The effects of FLI on MAPK and WNT are supported, as FGF2, LIF, and IGF1 each stimulate MAPK, and a crosstalk between MAPK and WNT signaling exists (Zhang & Liu 2002, Kuijk *et al.* 2012, Warzych *et al.* 2020). Thus, FLI supplementation improves embryo development and overall viability through stimulation of regulatory pathways that improve cell differentiation, proliferation, and survival.

In summary, supplementation of the FLI cocktail into bovine embryo culture medium at the beginning of culture can initiate changes in the embryo that are reflected temporally throughout preimplantation embryo development. These embryos may appear phenotypically similar, but at the level of the transcriptome, differences in message expression for transcripts involved in mechanisms such as Hippo and WNT signaling are apparent. In the FLI group, there was an increase in activation of TGF-β signaling at the morula stage, a decrease in WNT signaling at the blastocyst stage, and an alteration in MAPK signaling throughout development, thus revealing the effect of FLI on the fine regulation of embryo development. Collectively, these findings help elucidate the differences between embryos treated with and without FLI during *in vitro* culture and help improve understanding of mechanisms regulating embryo growth and viability.

## Supplementary materials





## Declaration of interest

The authors declare that there is no conflict of interest that could be perceived as prejudicing the impartiality of the work reported.

## Funding

This research is supported by the Clifton Murphy Scholarship Fund, USDA National Needs Fellowship funded by USDA NIFA Grant 2019-38420-28972, and USDA NIFA Predoctoral Fellowship Grant 2022-67011-36568.

## Author contribution statement

KM conceived the study, performed experiments, and wrote the paper. RP contributed to study design, results discussion, and final manuscript review. MSO contributed to the study design, analysis, and drafting of the manuscript.
